# Effect of Household Laundering, Heat Drying, and Freezing on the Survival of Dermatophyte Conidia

**DOI:** 10.3390/jof8050546

**Published:** 2022-05-23

**Authors:** Mohammad Akhoundi, Jade Nasrallah, Anthony Marteau, Dahlia Chebbah, Arezki Izri, Sophie Brun

**Affiliations:** 1 Parasitology-Mycology Department, Avicenne Hospital, AP-HP, Sorbonne Paris Nord University, 93009 Bobigny, France; jadenasrallah@gmail.com (J.N.); anthony.marteau@aphp.fr (A.M.); dahlia.chebbah@gmail.com (D.C.); arezki.izri@aphp.fr (A.I.); 2 Unité des Virus Émergents (UVE: Aix-Marseille Université-IRD 190-Inserm 1207-IHU Méditerranée Infection), 13005 Marseille, France

**Keywords:** dermatophytes, *Trichophyton*, decontamination, disinfection, freezing, laundering, heat drying

## Abstract

Dermatomycoses are one of the most common dermatological infectious diseases. Dermatophytoses, such as tinea pedis (athlete’s foot) in adults and tinea capitis in children, are the most prevalent fungal diseases caused by dermatophytes. The transmission of anthropophilic dermatophytoses occurs almost exclusively through indirect contact with patient-contaminated belongings or environments and, subsequently, facilitates the spread of the infection to others. Hygienic measures were demonstrated to have an important role in removing or reducing the fungal burden. Herein, we evaluated the effectiveness of physical-based methods of laundering, heat drying, and freezing in the elimination of *Trichophyton tonsurans, T. rubrum,* and *T. interdigitale* conidia in diverse temperatures and time spectra. Based on our findings, laundering at 60 °C was effective for removing the dermatophyte conidia from contaminated linens. On the contrary, heat drying using domestic or laundromat machines; freezing at −20 °C for 24 h, 48 h, or one week; and direct heat exposure at 60 °C for 10, 30, or 90 min were unable to kill the dermatophytes. These results can be helpful for clinicians, staff of children’s communities, and hygiene practitioners for implementing control management strategies against dermatophytoses caused by mentioned dermatophyte species.

## 1. Introduction

Dermatophytoses are the most common fungal infections in humans, caused by filamentous fungi. Anthropophilic species are the most common agents of dermatophytosis in humans. In developed countries, *Trichophyton rubrum* is the most prevalent dermatophyte, causing 70% of all anthropophilic dermatophytoses, notably in adults. It is responsible for over 80% of the athlete’s foot cases, followed by *T. interdigitale* [1]. In children, tinea capitis (TC) is the most frequent fungal infection and a widespread health problem all over the world. Over the past decade, TC of anthropophilic origin has significantly increased, particularly in urban areas, leading to epidemics in children’s communities [2,3,4].

Dermatophytes can spread in different ways, by direct contact to infected individuals, by indirect contact through skin scales or hairs left in the environment, or via the contaminated belongings of infected persons. Textiles in direct contact with affected skin areas or scalps are considered to be a major pathogen carrier [5]. Therefore, fomites are important players in the spread of dermatophyte infections. Dermatophyte propagules (cells or cellular elements serving dispersal) have an important role in the transmission of dermatophytosis in collective life, such as in households or children’s communities [6]. Factors such as lifestyle (e.g., sport club, swimming pools), close contact with infected individuals (e.g., contact sports or disadvantaged living conditions), and the exchange of contaminated fomites make inter-individual transmission more plausible [7]. Species such as *T. tonsurans* and *Microsporum audouinii* may be found on clothing and bedding, with the former also found on combs and brushes [7].

The treatment of dermatophytosis relies mainly on topical and/or oral antifungal medications depending on the location and extent of infection. Oral antifungal therapy (e.g., terbinafine, griseofulvin, or itraconazole) is the gold standard for treatment of TC and for proximal or multiple onychomycosis [8,9]. However, adjunctive treatment with topical antifungals, especially in TC, is necessary to limit conidia dissemination in an environment of infected individuals. Moreover, general protective measures are essential in reducing the risk of intra-family or intra-community dermatophyte transmission and in breaking the chain of infection. These measures include effective hygienic practices, handwashing, keeping feet clean and dry, keeping nails well-groomed, prompt showering after contact sports, and avoiding the sharing of personal hygiene items. Moreover, individuals with TC should disinfect brushes, combs, and clippers with antifungal powders.

Hygienic measures such as laundering are demonstrated to have an important role in removing or reducing the fungal burden of infected linens [5,10]. Given the importance of the mentioned measures in preventing dermatophytosis dissemination, and in spite of several informal guidelines recommending them for disease management, their effect on the survival of dermatophyte propagules under various time and temperature conditions has not been extensively investigated. Although they were the subject of some in vitro investigations, the examined conditions did not fully reflect natural conditions [10,11,12]. Furthermore, physical factors such as freezing or heating were shown to possess antimicrobial activity, which could influence the survival of dermatophytes [13]. The main goal of this study was to evaluate the lethal temperatures and times required for the chemical-free eradication of dermatophytes on linens by laundering, heat drying, or freezing in various situations, simulating a domestic setting. 

## 2. Materials and Methods

### 2.1. Isolates and Culture Conditions

*Trichophyton tonsurans* (from TC) and *T. rubrum* (from tinea pedis) isolates, recently identified in the Parasitology-Mycology Department of Avicenne hospital (Bobigny, France) together with one reference strain of *T. interdigitale* (ATCC 9533), were used for all assays. The two clinical isolates were identified at the species level by macroscopic and microscopic analyses of fungal cultures and further confirmed by MALDI-TOF mass spectrometry using the MSI database (https://msi.happy-dev.fr/, accessed on 3 May 2021). The morphological identification of the clinical isolates was confirmed by conventional PCR targeting the ribosomal ITS region (ITS-rDNA) and bidirectional sequencing [14]. The latter was carried out using the same primers (ITS1: 5′-TCCGTAGGTGAACCTGCGG-3′ and ITS4: 5′-TCCTCCGCTTATTGATATGC-3′) used for PCR amplification. The sequences were deposited in GenBank under the assigned numbers of NX9120497 and NX9120498. Three aforementioned strains (one strain per species) were used for all bioassays. The dermatophytes were inoculated with a sterile swab on the entire surface of 90 mm diameter Petri dishes containing Sabouraud glucose agar (SGA) (Oxoid, Basingstoke, UK), with gentamycin (0.1 g/L) plus chloramphenicol (0.05 g/L) but without cycloheximide, and incubated at 27 °C for 2 weeks. Multiple sets of sterile 7.5 × 7.5 cm cotton gauze pads (Sylamed, Paris, France) were divided into three groups (according to the number of dermatophyte species tested) and contaminated by gentle short presses on the SGA plates containing each dermatophyte colony culture. Each contaminated gauze pad was then stored in a sterile Petri dish to be used immediately for bioassays.

### 2.2. Laundering and Heat Drying 

Each assay of laundering and heat drying was performed separately for the three mentioned species in triplicate and in three independent experiments. One sterile gauze pad contaminated with each dermatophyte species was kept at ambient room temperature as a positive control for each experiment. Non-contamination of laundering and heat drying machines was ensured by examining the sterile gauze pads as controls before each experiment. 

Labeled gauze pads previously contaminated with *T. tonsurans*, *T. rubrum*, or *T. interdigitale* were individually placed within tissue-meshed mini-pockets and laundered in a domestic laundering machine (WAB24211FF Bosch, München, Germany) at diverse temperatures (40, 60, or 90 °C) and times (100 or 135 min) in two different ways: (i) using domestic detergent (Eveil Floral liquid hypoallergenic, L’Arbre Vert, Cavaillon, France) recommended for domestic use, or (ii) without detergent. This detergent was composed of 5 to 15% anionic and nonionic surfactants, separately, less than 5% soap, polycarboxylates, enzymes such as amylases and proteases, as well as perfume. 

The effect of heat drying was evaluated by placing previously contaminated gauze pads containing the aforementioned dermatophyte species in a domestic drying machine (PerfectCare 700, Electrolux, Stockholm, Sweden) for 100 and 150 min or in laundromat drying machines (coin-operated dryer machine for public use, T5300S, Electrolux) for 10 min. 

After laundering or heat drying, each individually processed gauze pad was incubated at 27 °C on an SGA plate for three weeks and monitored twice a week for possible growth of dermatophytes. Dermatophyte identities were verified by the morphological analysis of the cultures [15].

### 2.3. Direct Exposure to Freezing and Heating

The labeled cotton gauze pads previously contaminated by *T. tonsurans*, *T. rubrum*, or *T. interdigitale* were exposed separately at −20 °C (Liebherr G 5216-21, Kirchdorf an der Iller, Germany) for 24 h, 48 h, or 1 week, and at 60° C (laboratory incubator BD115, Binder, Tuttlingen, Germany) for 10, 30, or 90 min. Like previous bioassays, the processed pads were incubated on SGA plates at 27 °C and monitored for three weeks for possible growth of dermatophytes.

The temperatures during each of the aforementioned experiments were monitored by a temperature data logger (21G ProgresPlus, Willems, France).

## 3. Results

A total of 180 cotton gauze pads contaminated by *T. tonsurans*, *T. rubrum*, or *T. interdigitale* were separately subjected to laundering (54 pads), heat drying (27), freezing (27), or direct heat exposure (27). The detailed results of the mentioned bioassays are given in Table 1 and Table 2. 

No dermatophyte growth on SGA was observed for contaminated gauze pads laundered at 60 °C or 90 °C for 100 or 135 min, respectively, while all of the contaminated pads washed at 40 °C for 100 min were positive for dermatophytes within a few days of incubation on SGA plates. Similar results were obtained for all assays performed with or without detergent (Table 1). 

Regarding heat drying assays using domestic or laundromat machines, all SGA plates inoculated with the processed pads were positive for dermatophytes within a week (Table 1). 

All cultures of contaminated gauze pads exposed to freezing (−20 °C) for 24 h, 48 h, or 1 week and those processed by direct heat exposure at 60 °C for 10, 30, or 90 min were positive within a few days of incubation on SGA plates (Table 2). 

For the 45 positive control gauze pads kept at laboratory ambient temperature, SGA plates were positive for dermatophytes in less than a week.

## 4. Discussion

Dermatophytes are the most common fungi causing superficial infections of the skin, hair, and nails. People are usually sensitized to the risk of dermatophyte infections in public places such as schools, nurseries, swimming pools, and sport clubs. Due to the high prevalence, recurrence, and reinfection rates associated with dermatophyte infections, the need for efficient preventive strategies is evident [16,17]. Laundering is a sanitization method that has been reported to remove dermatophyte conidia from clothing and linens. The proper use of this method eliminates or reduces not only the recurrence and relapse rates of dermatophytoses, but also decreases the economic burden associated with these infections [18]. In return, improperly laundered linens may harbor dermatophytes and facilitate infection spread [5,19]. Therefore, the conditions for washing clothes are important, especially for domestic use. Temperature and time are among the most important factors that are involved in effective elimination of dermatophyte propagules via laundering [10]. 

Although laundering has been recommended in several informal guidelines as an effective way to remove dermatophyte conidia, they suffer from a lack of consensus on laundering conditions (e.g., temperature and duration) [20,21]. In France, a single guideline published by the Ministry of Health recommends handwashing and cleaning toys in case of dermatophytoses in a community, without giving any recommendation on laundry decontamination [22]. Therefore, there is no reliable recommendation for disinfecting clothes and household linens contaminated by conidia of dermatophytes. 

**Figure 1:**
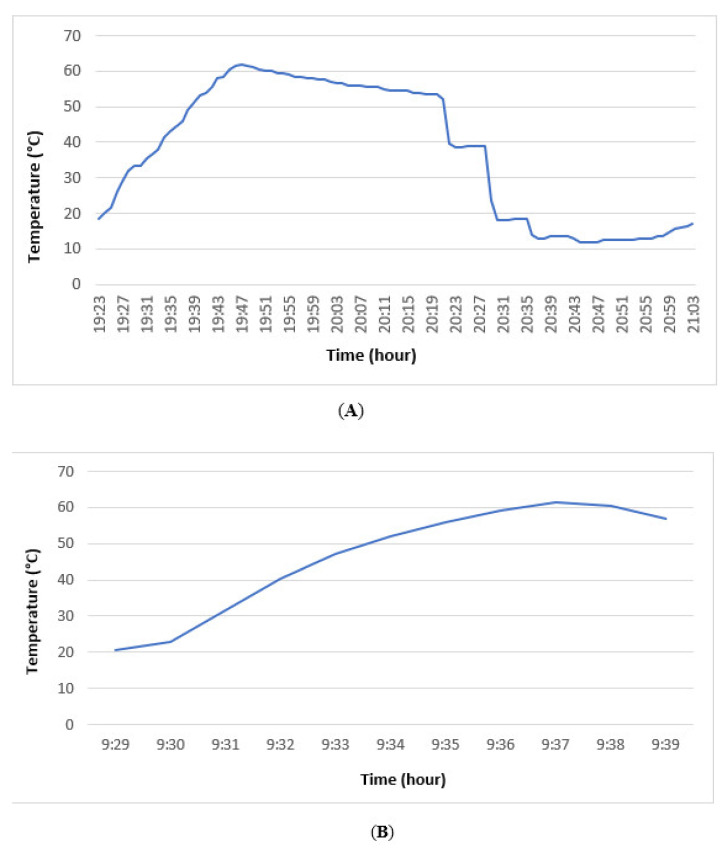
(**A**) Temperature courses of the laundering program at 60 °C for 100 min using domestic machine; (**B**) temperature variations during heat drying performed using laundromat.

In this study, we assessed whether domestic laundering or heat drying were suitable in removing dermatophyte conidia from linens. Therefore, we evaluated the effectiveness of these non-chemical treatments of contaminated gauze pads in terms of qualitative dermatophyte growth (ability for total elimination or not of dermatophyte growth) and not quantitative growth comparison. Since this study was conducted based on qualitative evaluation, no quantitative determination was performed. Cotton gauze pads previously contaminated by *T. rubrum*, *T. tonsurans*, or *T.* *interdigitale* were washed using a domestic laundering machine at diverse temperatures of 40, 60, and 90 °C, with or without detergent. Cotton gauze pads were similar in texture to most cotton-made linens and easy to apply to Sabouraud agar plates due to their small size. They were contaminated with a very high burden of conidia that was significantly higher than conidia levels in the environment of an infected patient. Based on our results, washing linens at 60 °C is essential for the successful removal of dermatophyte conidia. Surprisingly, temperature monitoring during laundering at 60 °C revealed that this temperature was only achieved during 8 out of 100 min (Figure 1A). The effect of domestic laundry processes on dermatophytes was evaluated by Ossowski [19], who reported the effectiveness of laundering using detergent at 30 °C for eliminating *T. rubrum* conidia, while *T. mentagrophytes* was eliminated only at 60 °C. Conversely, Hammar et al. [5] stated the ineffectiveness of washing *T. rubrum*-contaminated linens at 30 °C, while washing at 60 °C was highly efficient in eliminating dermatophyte conidia. Afterwards, the ineffectiveness of laundering at 40 °C for eradicating *T. rubrum* was reported by Amichai et al. [10]. Therefore, in spite of current recommendations for energy saving and environmental protection, laundering at low temperatures was not effective in eradicating dermatophyte propagules and it may increase the risk of contamination. On the other side, conventional laundering without detergent was reported to be inefficient in removing fungal conidia [23]. Based on our findings, no difference was observed since all samples laundered with or without detergent at 60 °C resulted in the elimination of dermatophyte conidia. 

To the best of our knowledge, no investigation has been conducted on effect of heat drying on dermatophyte viability in the literature. Based on our findings, heat drying was unable to eliminate dermatophyte conidia using domestic or laundromat machines. The temperature variations recorded by the data logger in the domestic dryer revealed that the temperature remained stable during the drying process at an ambient temperature of 20 °C for 150 min. Concerning laundromat heat dryers, a gradual increase in temperature was observed during 10 min of the drying program with only 2 min at 60 °C (Figure 1B). The inability to reach a high temperature up to 60 °C or too short of a duration can partly explain the ineffectiveness of heat drying in killing dermatophyte conidia in our bioassays.

Physical-based methods demonstrated a significant impact on the development and survival of dermatophytes [24]. One of the first assays of freezing on the dermatophytes was carried out over 60 years ago, demonstrating the successful effect of freezing for dermatophytes (*T. violaceum, T. acuminatum, T. rubrum*, and *Microsporum canis*) conservation [25]. A longer period of dermatophyte viability was reported after freezing for over 20 years [26,27]. Hasegawa [28] examined the viability of *M. canis* at freeze-dried conditions (−20 °C, −80 °C, and in liquid nitrogen) and reported the preservation of *M. canis* within 24 h and 1-month freeze drying. Deshmukh [29] tested the freeze-dried cultures of keratinophilic fungi and related dermatophytes after 12 years with no loss of viability. These results are consistent with our findings certifying the high resistance of *T. rubrum, T. tonsurans* and *T. interdigitale* to freezing. Therefore, freezing can be served in the short term or for long-term dermatophytes preservation. 

The effect of direct heat exposure on dermatophyte survival was the subject of only a few investigations in the literature. Sinski et al. [12] reported no significant difference between *T. mentagrophytes* colony counts obtained from clinical specimens exposed to 60 °C for up to four hours and unexposed controls. In an investigation carried out on the heat resistance of dermatophytes isolated from Nigerian athlete’s kits, *Epidermophyton floccosum*, *M. audouinii*, *M. canis*, *T. concentricum*, *T. mentagrophytes*, or *T. rubrum* conidia in Sabouraud dextrose agar broth exhibited high resistance to heat treatment at 80 °C up to 28 min [30]. In another assay performed by the same authors, *Microsporum* sp., *T. rubrum*, *T. mentagrophytes*, and *Epidermophyton floccosum* on SGA plates survived after exposure to 90 °C for 2 weeks [31]. 

The direct heat exposure of contaminated gauze pads at 60 °C for 10, 30, or 90 min in the present study revealed the ineffectiveness of heat exposure in inhibiting the growth of dermatophytes. Based on these results and according to the Sinner concept [32], temperature, time, mechanical action, and chemistry are the parameters influencing the antimicrobial efficacy of laundering. Temperature is one of the most important factors for ensuring laundry hygiene. Regarding the ineffectiveness of direct heat exposure in removal of dermatophyte conidia, it seems that, along with temperature, there are other agents playing an essential role in the antimicrobial efficacy of the laundering process. Due to the lack of differences in laundering results with and without detergent, it seems that repeated rinsing with water discharge during laundering is a determining factor in eliminating the dermatophyte conidia. This hypothesis is supported by a recent investigation stating that, in washing cycles with low temperatures and low amounts of detergent, the mechanical action might have an important effect resulting in the physical removal of microorganisms from textiles [33].

## 5. Conclusions

The evaluation of physical-based methods of laundering, heat drying, and freezing in the elimination of dermatophytes from previously contaminated gauze pads at diverse exposure times and temperatures allowed us to highlight the effectiveness of laundering at 60 °C in the removal of conidia of T. tonsurans, T. rubrum, and T. interdigitale. Conversely, heat drying using domestic or laundromat machines was inefficient in eliminating dermatophyte conidia. These findings can be useful for clinicians, staff of children’s communities, and hygienic practitioners to incorporate into control management strategies of dermatophytoses.

## Figures and Tables

**Table 1:** Viability of cells/conidia of three *Trichophyton* species on previously contaminated gauze pads after laundering or heat drying at diverse temperatures and times.

Dermatophyte Species	Laundering						Heat Drying		
	With Detergent			Without Detergent			Domestic Machine		Laundromat
	40 °C 100 min	60 °C 100 min	90 °C 150 min	40 °C 100 min	60 °C 100 min	90 °C 150 min	100 min	150 min	10 min
*Trichophyton tonsurans*	+	-	-	+	-	-	+	+	+
*Trichophyton rubrum*	+	-	-	+	-	-	+	+	+
*Trichophyton interdigitale*	+	-	-	+	-	-	+	+	+

(+): growth of dermatophyte on Sabouraud glucose agars incubated for three weeks at 27 °C; (-): absence of dermatophyte growth.

**Table 2:** Viability of cells/conidia of three *Trichophyton* species on previously contaminated gauze pads after freezing and direct heat exposure at diverse temperatures and times.

Dermatophyte Species	Freezing (−20 °C)			Heating (60 °C)		
	24 h	48 h	1 week	10 min	30 min	90 min
*Trichophyton tonsurans*	+	+	+	+	+	+
*Trichophyton rubrum*	+	+	+	+	+	+
*Trichophyton interdigitale*	+	+	+	+	+	+

(+): growth of dermatophyte on Sabouraud glucose agars incubated for three weeks at 27 °C.

## Data Availability

Not applicable.
